# The value of primary transcripts to the clinical and non‐clinical genomics community: Survey results and roadmap for improvements

**DOI:** 10.1002/mgg3.1786

**Published:** 2021-08-26

**Authors:** Joannella Morales, Aoife C. McMahon, Jane Loveland, Emily Perry, Adam Frankish, Sarah Hunt, Irina M. Armean, Paul Flicek, Fiona Cunningham

**Affiliations:** ^1^ European Molecular Biology Laboratory European Bioinformatics Institute, Wellcome Genome Campus, Hinxton Cambridge United Kingdom

**Keywords:** default transcript, survey, transcript annotation, variant interpretation

## Abstract

**Background:**

Variant interpretation is dependent on transcript annotation and remains time consuming and challenging. There are major obstacles for historical data reuse and for interpretation of new variants. First, both RefSeq and Ensembl/GENCODE produce transcript sets in common use, but there is currently no easy way to translate between the two. Second, the resources often used for variant interpretation (e.g. ClinVar, gnomAD, UniProt) do not use the same transcript set, nor default transcript or protein sequence.

**Method:**

Ensembl ran a survey in 2018 to sample attitudes to choosing one default transcript per locus, and to gather data on reference sequences used by the scientific community. This was publicised on the Ensembl and UCSC genome browsers, by email and on social media.

**Results:**

The survey had 788 responses from 32 different countries, the results of which we report here.

**Conclusions:**

We present our roadmap to create an effective default set of transcripts for resources, and for reporting interpretation of clinical variants.

## Graphical abstract

After decades of avoiding the demand to highlight one transcript per locus in Ensembl, we ran a survey 2018 to assay opinions across the scientific community. Ignoring the problem of ‘one transcript’ was not making the issue go away; many important genomic resources had instead adopted their own methods of selecting one transcript (e.g. HGMD, Ensembl, gnomAD, UniProt, ClinVar, etc.). Here we report our results and roadmap to create an effective default set of transcripts for resources, and for reporting interpretation of clinical variants.

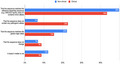

## 1 INTRODUCTION

Many advances in biological understanding and genomic medicine are dependent on variant interpretation and the ability to describe a sequence change with respect to a specific annotated transcript. However, in publications the transcript information required to reuse data is only very rarely recorded accurately, hampering the ability to reuse the data. For example: (a) unspecified, and sometimes historical, transcripts have frequently been used (e.g. CFTR del‐508, BRAF V600E); (b) despite the existence of Human Genome Variation Society (HGVS) guidelines for variant reporting (den Dunnen et al., 2016), no transcript version is specified; (c) legacy numbering is commonly provided: for example for proteins with a signal peptide, the numbering can begin downstream after the signal peptide, so amino acid 1 is not the usual methionine.

Moreover, interpretation of novel data is hampered by the variety of reference sequences used to gather evidence for variant analysis, and lack of coordination across the resources. There are two commonly used transcript sets for annotation: NCBI’s RefSeq (O’Leary et al., 2016) and EMBL‐EBI’s Ensembl/GENCODE (Frankish et al., 2021). Many highly‐accessed genomics resources supporting variant interpretation use transcripts from only one set, or default to a single transcript (e.g. ExAC/gnomAD (Karczewski et al., 2020; Lek et al., 2016), Human Cell Atlas (Andersson et al., 2014), GTEx (GTEx Consortium et al., 2015), ClinVar (Landrum et al., 2014), HGMD (Stenson et al., 2020). None of these are coordinated with UniProt's principal isoform (Bateman et al., 2017) and comparison of annotation across sets is non‐trivial. Additionally, some transcript sequences do not perfectly match the reference genome used for variant calling.

With this in mind, we started to explore how to choose one default transcript for each protein‐coding locus, and the merits of such a set. In 2018, we surveyed the community to understand the priorities and attitudes surrounding transcript choice and reporting. The survey results supported RefSeq and Ensembl/GENCODE agreeing on an identical transcript for each locus to be used as a common default across resources. Below we detail our other conclusions.

## 2 METHODS

To gather input from the scientific community on transcript usage, and attitudes to transcript change, we developed a survey (see supplementary file). The survey had four sections: ‘Transcript choice’, ‘Variant interpretation and reporting’, ‘Reference sequence sources’, and one on the demographics of the respondents. We had compulsory questions that required selection of a single answer, and optional questions that were a mixture of multiple‐choice questions and open‐ended questions. For example our questions covered:

‐ What the demand was for a single transcript per locus, a minimal set of transcripts or a complete set of all known transcripts. For the minimal set, whether that should cover all exons with clinical significance, or all abundant protein‐coding exons, or all abundant exons.
‐ How to choose one primary transcript per locus, raising awareness of the complexities and compromises when selecting one transcript. We had a series of questions where the respondent had to trade‐off: low abundance and longer coding sequence with higher abundance and a shorter coding sequence; or abundance, coding sequence length and coverage of clinically relevant variants.
‐ The relative importance of transcripts remaining stable, or matching the reference assembly, or avoiding pathogenic alleles or including globally frequent alleles.
‐ Opinions on updating a transcript to change the coding sequence, UTR length, transcript splicing or never updating.
‐ The reference sequences currently used, including for interpreting and reporting variants.
‐ The value of having different transcripts sets versus having increased agreement between RefSeq and Ensembl/GENCODE.

The examples we chose for picking transcripts were cartoon versions of real loci. We advertised the survey by email, on the Ensembl (Howe et al., 2021) and UCSC (Tyner et al., 2017) genome browsers, via social media, and through contacts to ClinGen and NCBI’s Genetic Testing Registry participants.

## 3 RESULTS

The survey generated 788 responses (see supplementary file for questions and results and https://tinyurl.com/embl‐ebi‐transcript‐survey) from 32 different countries: the largest contributors were the USA, UK and Germany (40%, 19% and 5% respectively). Not all respondents answered every question as some were optional. We assayed how transcripts were used across the scientific community (question 14). The most common words in the free‐text answers included: variants, analysis, expression, RNA‐seq, clinical, reporting, gene and annotation.

We analysed our results in two categories based on the response to the multiple‐choice question ‘Where do you work?’. Those who selected ‘clinical diagnostics’ or ‘clinical research’ were labelled ‘clinical’ (*N* = 285; 36%) and those who selected from (‘University/college/academia/non‐profit/research’; ‘commercial/industry; government’; ‘other’) were ‘non‐clinical’ (*N* = 503; 64%). For those involved in clinical reporting of variants, done via clinically accredited pipelines, we assumed the requirements were for data consistency between patients. Therefore, updates to a resource, software, or gene assembly that require pipeline re‐accreditation and remapping of large internal datasets would be a challenge in a manpower‐stretched clinical laboratory. Contrastingly, the requirements for the non‐clinical, researcher‐based category, would be to have the latest toolset and to use the most recent research data for analysis. We wanted to see if this requirement difference manifested in the results.

**FIGURE 1:**
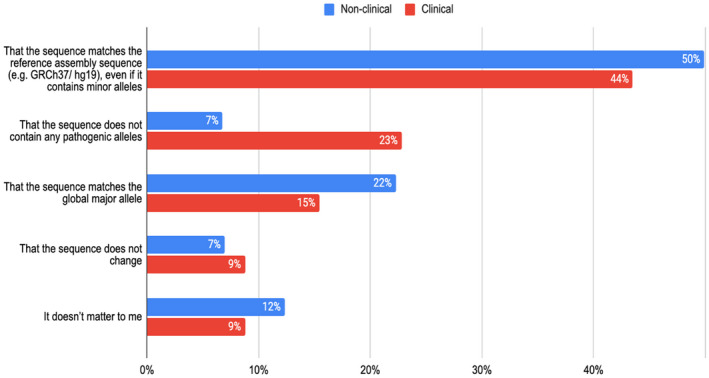
An example of a cartoon version of a locus we used in the survey to understand opinions across the scientific community on different options for choosing one transcript. These are the transcript scenarios presented for questions 2a (top panel) and 2b (bottom panel). For question 2a, and for question 2b, we asked respondents to choose either the first longer coding transcript, or the second more abundant (but shorter) one as a primary transcript. For both questions, the more abundant one (indicated by the blue arrow) was the most popular transcript choice for the non‐clinical community (75%; 68%). However, there was no clear preference for this one (indicated by the blue arrow) from the clinical respondents (54%; 46%).

**FIGURE 2:**
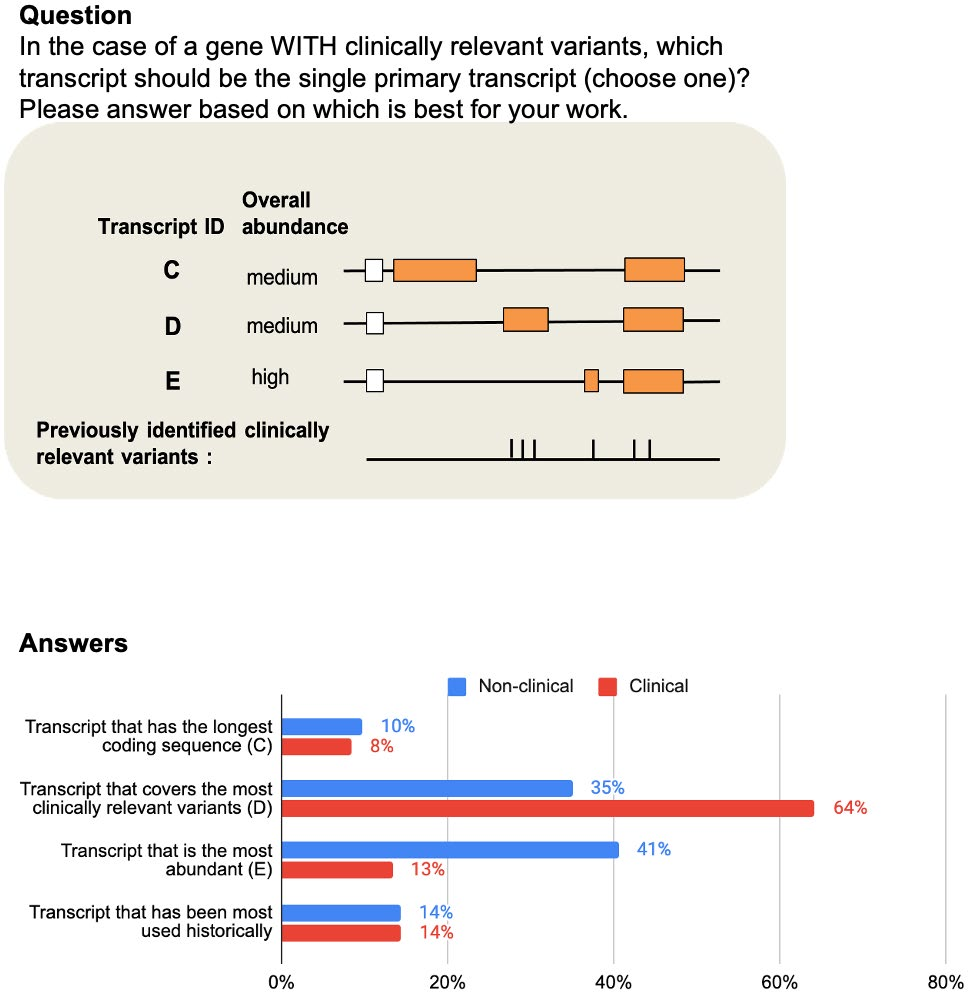
Top panel: question 3a from the survey. Bottom panel: bar chart of answers across 503 ‘non‐clinical’ respondents and 285 ‘clinical’ ones. Respondents chose between the transcript that has the longest coding sequence (C), that covers the most clinically relevant variants (D), that is most abundant (E) or that is used historically. The results favoured (D), the transcript that covers the most clinically relevant variants, or (E) the most abundant overall. However, for the clinical group, there was a strong preference for (D) the transcript that covers the most clinically relevant variants (64%) despite having lower abundance overall. In contrast, there was no obvious preference between these choices for the non‐clinical group. Here neither the longest coding transcript (C), nor the historical transcript were popular preferences.

When presented with the choice between a more abundant transcript or a transcript with a longer coding sequence for the primary transcript, the non‐clinical group showed a clear preference for choosing the more abundant transcript, with 75% and 68% of respondents choosing this option in questions 2a and 2b respectively. In contrast, no clear preference emerged in the clinical group (see Figure 1). In question 3a, the choice was between the transcript that covers the most clinically relevant variants, or that is most abundant, or that is longest, or that is used historically. The clinical group preferred the transcript that covered the most clinically relevant variants (64%, see Figure 2); (see also question 3b). In contrast, there was no obvious preference between these choices in questions 3a, 3b for the non‐clinical group. There was low preference in both categories for historical transcripts (12%; 14% of respondents—question 3a; 3b).

We received >800 additional free‐text comments across questions 1–3. Themes that emerged from these: rejected the value of a primary transcript, stated that all transcripts should be used, or proposed an artificial transcript be created to cover all exons. Many comments called for ranking and filtering methods in genome browsers and resources, supported by specific data on transcript abundance, tissue‐specificity/expressivity, cell‐specificity, background conditions, environmental, developmental stage and transcript quality metrics. More data were requested on flagging transcripts that were computationally determined, predicted, fully functional, validated, chosen by expert consensus as clinically relevant, or rare. The importance of cell/tissue‐specificity and the difficulty of assessing abundance or relative expression was often mentioned.

**FIGURE 3:**
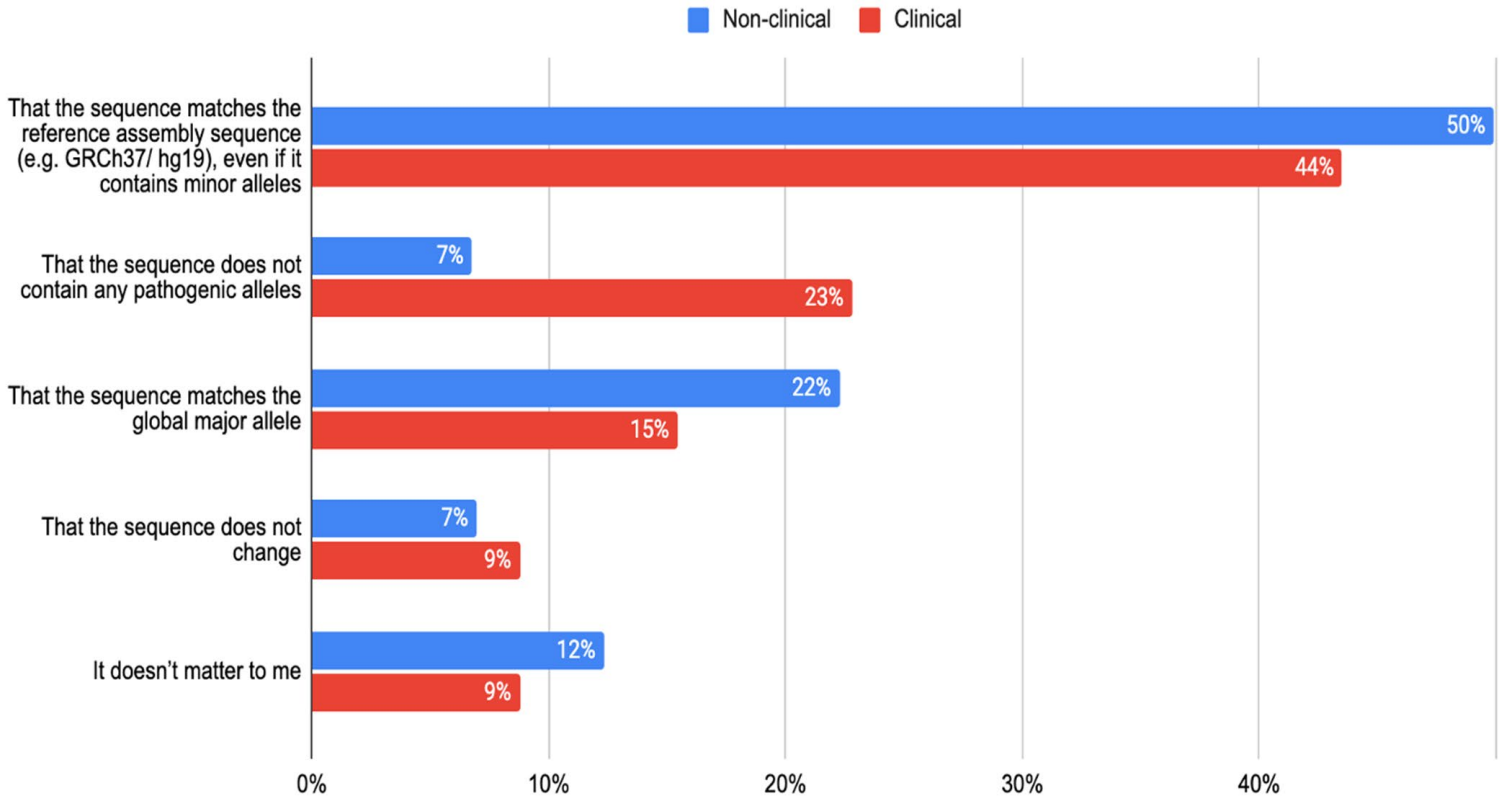
Bar chart of results from question 4 which asked ‘Considering the sequence of a transcript, which is the most important to you (choose one): that the sequence matches the reference assembly sequence (e.g. GRCh37/hg19), even if it contains minor alleles; that the sequence does not contain any pathogenic alleles; that the sequence matches the global major allele; that the sequence does not change; or it does not matter to me. Both the clinical (*N* = 285) and non‐clinical (*N* = 503) respondents had ‘that the sequence matches the reference’ as most important (44%; 50%). For many in the clinical group, however, it was also important that a transcript did not contain any pathogenic alleles (7% of ‘non‐clinical’ respondents but 23% ‘clinical’ ones). Only a minority prioritised that a transcript sequence never changes (<10%).

For transcript sequences, (in question 4) respondents were asked to prioritise that either a transcript sequence matches the reference assembly, does not contain pathogenic alleles, matches the global major allele or never changes. Here, the transcript that matches the reference was the priority choice (48%) across all respondents (Figure 3). There was only a minority to whom transcript sequences never changing was important (<10%, questions 4 and 5).

For variant interpretation and reporting in question 6, there was a preference captured across all respondents for ‘I wouldn't use just one transcript for INTERPRETATION unless it was the only one known’ (77%) over only using one transcript (23%). The preferred option for clinical respondents was to report on the primary transcript and the affected transcript (39%) rather than across all transcripts (14%). The opposite was true for the ‘non‐clinical’ group (18% vs. 40% respectively; question 7).

**FIGURE 4:**
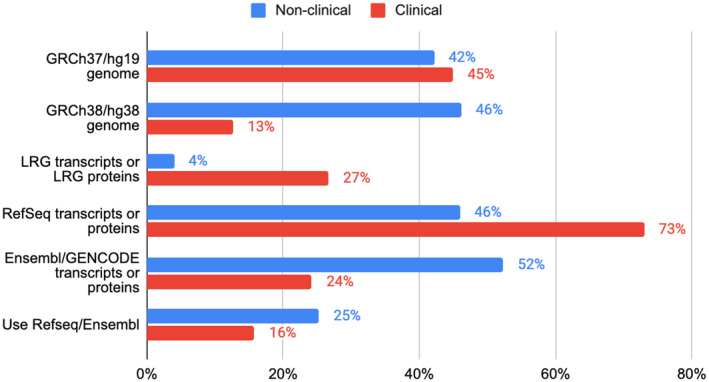
Answers across respondents (503 ‘non‐clinical’ and 285 ‘clinical’) for usage of both the genome build choice and the transcript set. Question 8 asked ‘Which reference sequences do you use for reporting variants (select all that apply)’: GRCh37/hg19 genome; GRCh38/hg38 genome; LRG transcripts or LRG proteins; RefSeq transcripts or proteins; Ensembl/GENCODE transcripts or proteins. In general, the ‘clinical’ respondents used GRCh37/hg19 (45%) rather than GRCh38 (13%) as a reference assembly, RefSeq transcripts or proteins rather than Ensembl/GENCODE (73% vs. 24%), and LRG transcripts or proteins (27%). Whereas the ‘non‐clinical’ community replies were more equally spread across using GRCh37 and GRCh38 (42% vs. 46%) for reference assemblies, RefSeq or Ensembl/GENCODE (46% vs. 52%), and little usage of LRG (4%).

We surveyed the reference sequences used for reporting in question 8 (Figure 4). In general, ‘clinical’ respondents used RefSeq (73%), Locus Reference Genomic (LRG), 27% (Dalgleish et al., 2010; MacArthur et al., 2014) and GRCh37 (71%), rather than Ensembl/GENCODE (24%) or GRCh38 (19%). Whereas the ‘non‐clinical’ community replies were more equally spread across using GRCh38 (46%) and GRCh37 (42%), RefSeq (46%) or Ensembl/GENCODE (52%) but not LRG (4%).

Results from the survey indicated that having RefSeq and Ensembl/GENCODE agree on one primary transcript per gene would be welcome (54% overall; 67% of ‘clinical’ respondents, question 10). We revisited the question ‘Do you want us to provide one primary transcript’ (‘Question 1 revisited—a primary transcript’) after question 14 at the end of the survey requiring a ‘Yes’, ‘No’ or ‘Not sure’ answer. Here 60% of the ‘clinical’ respondents were in favour, compared with 48% of ‘non‐clinical’ ones.

With input from these survey results, our conclusions and recommendations are that:

1. RefSeq and Ensembl/GENCODE collaborate to agree on:
  - one identical primary transcript per locus that perfectly matches the GRCh38 reference assembly. This is to ensure the community, browsers and resources use a good, consensus choice of transcript for analyses or situations that require only one (e.g. default display per gene).
  - minimal additional identical transcripts that match the reference assembly, which are required for clinical reporting.
2. Transcripts are updated from historical exemplars, using modern datasets to choose a representative transcript:
  - evaluated on predicted functional significance and abundance rather than due to longest length, or being defined first (i.e. the historical transcript).
  - whose sequence is an exact reference genome sequence match.
3. All resources adopt this primary agreed transcript for the most effective benefit of the workings of the scientific community.
4. Genome browsers and resources consider improvements to their methods of filtering and ranking transcripts to facilitate choosing the appropriate transcript(s). Often, using only the one primary transcript per locus may not be right.

We have used these recommendations to guide our collaborative work with RefSeq on the Matched Annotation from NCBI and EMBL‐EBI (MANE) collaboration (see http://tark.ensembl.org/web/mane_project/; https://www.ncbi.nlm.nih.gov/refseq/MANE/).

## 4 DISCUSSION

Across the survey results as a whole, there is no single preferred method for designating a primary transcript. However, achieving consensus between Ensembl/GENCODE and RefSeq on a primary transcript was highlighted as highly valuable. There is a history of collaboration between the two groups, for example on the Consensus CDS (CCDS) project (Pujar et al., 2018) and LRG. For many transcripts, the CCDS project has achieved consensus for the exon/intron structure over the protein‐coding region, but there remains coding sequence discrepancies and structure differences in the untranslated regions (UTRs). The LRG project focuses on recording historical sequences for variant reporting that will never change, and many of these sequences do not perfectly match the reference assembly or use the latest evidence. However, the survey demonstrated a tolerance for change (only 6% selected ‘Never update’ in question 5).

Interestingly, many respondents suggested the ideal primary transcript should contain all exons. This ‘meta transcript’ approach has been used for a few LRGs (e.g. LRG_391 for TTN; and LRG_202 for NEB) to represent an inferred transcript model containing all identifiable in‐frame coding exons. However, it leads to the creation of primary transcripts that do not reflect biological reality and which are not guaranteed to be comprehensive: they may contain exons that show huge differences in their inclusion rates generally, and are tissue‐specific; they may include mutually exclusive exons; they cannot include exons in different frames and they will need to be updated if novel coding exons are subsequently discovered.

The survey reported many, especially clinical groups, are still using GRCh37, released in 2009. GRCh38, released in 2013, offers a more complete genome that is being continuously improved by the Genome Reference Consortium (GRC; Schneider et al., 2017) through a supplemental release model. Ensembl/GENCODE gene annotation is only being updated on GRCh38. Therefore, it is only the annotation on GRCh38 that will benefit from all the improvements supported by the incorporation of new datasets (such as long transcriptomic data generated using methods developed by Oxford Nanopore Technologies and Pacific Biosciences), and of tools (such as the PhyloCSF method (Lin et al., 2011) for identifying regions of the genome with conserved protein‐coding potential). Major resources such as gnomAD and DECIPHER (Firth et al., 2009) are also now using GRCh38.

Worth noting is that many survey comments expressed resistance to the very idea of a default transcript. They rightly pointed out that biology cannot be simplified in this manner, however appealing the concept. We agree completely that genome analysis requires consideration of multiple transcripts per gene and Ensembl remains absolutely committed to annotating all evidence‐based transcripts at every locus. Analysis, including the interpretation of variants identified from clinical sequencing, should always be in relation to the most relevant and abundant isoform(s) for the tissue of interest at the relevant developmental stage and in the correct cell type. In general however, we do not yet have the data to determine this. Although projects such as GTEx and Human Cell Atlas have and will change the landscape of transcriptomic data available, currently for most developmental stages, there is a lack of this critical information. As a result, in the absence of tissue‐specific data, any analysis should consider all transcripts or proteins at the locus. We urge more cooperation between clinical diagnostics and research to use a broader transcript set and thereby remove the bias in reported transcripts.

However, for practical reasons, it is sometimes helpful to have only one transcript for sharing and comparing results across experiments, datasets and collaborations. Indeed, many browsers, bioinformatics tools and variant interpretation pipelines have chosen a default transcript, independently from each other. For example Ensembl and UniProt have had their own ‘canonical’ (available only through the Ensembl API) and ‘principal isoform’ choices for default transcripts and proteins respectively, for over a decade. RefSeq has a ‘select’ transcript and HGMD has a default RefSeq. Often these choices have been based on the longest transcript, or the first sequences published, or most prevalent (https://www.uniprot.org/help/canonical_and_isoforms) but are not necessarily consistent or agreed with other resources.

It is clear, therefore, that the concept of a default transcript already exists across resources but is uncoordinated. The survey results demonstrate a desire for a default transcript, but in the absence of a consensus choice so far, we see different default transcripts chosen for different studies and genomics resources. Selecting one particular transcript per locus comes with a risk of biasing the scientific community towards ignoring the full transcriptome. However, our collaboration between RefSeq and Ensembl/GENCODE will provide the leadership necessary to unite the community and provide a consensus choice, which the survey shows is currently lacking. This will be a practical and coordinated effort to define one default transcript per locus. There is no overall ‘correct’ choice for a default transcript. Most important and valuable is consistency for reporting and to ease use across resources and tools that require a default transcript. Equally important will be to work with all major browsers and resources (e.g. NCBI, Ensembl, the Ensembl Variant Effect Predictor, UCSC Genome Browser, gnomAD, DECIPHER, UniProt, Panel App, COSMIC, etc.) to ensure adoption of the common default transcript.

## CONFLICTS OF INTEREST

PF is a member of the scientific advisory boards of Fabric Genomics, Inc., and Eagle Genomics, Ltd.

## AUTHOR’S CONTRIBUTIONS

Joannella Morales: survey design, survey analysis, survey promotion, manuscript input. Aoife C. McMahon: survey design, survey design feedback, survey figures, manuscript input. Jane Loveland and Adam Frankish: survey design, survey design feedback, manuscript input. Emily Perry: survey review, survey promotion, social media coordination, manuscript review. Sarah Hunt: bioinformatics analysis, manuscript review. Irina M. Armean: bioinformatics analysis, manuscript review. Paul Flicek: survey dissemination—twitter; survey design feedback, manuscript input. Fiona: wrote this manuscript, survey design, survey analysis, survey promotion.

## Supporting information

Supplementary Material

Supplementary Material

## Data Availability

The datasets generated and/or analysed during this study are available here. https://tinyurl.com/embl‐ebi‐transcript‐survey.
