# Supplementary material for: The value of primary transcripts to the clinical and non‐clinical genomics community: Survey results and roadmap for improvements
Source: Mol Genet Genomic Med. 2021 Aug 26;9(12):e1786. doi: 10.1002/mgg3.1786 (PMC8683622; doi:10.1002/mgg3.1786)
Supplement: Supplementary file 1 — Supplementary Material [file MGG3-9-e1786-s001.pdf]

Survey questions and results

Transcript Survey (one page)

Genes/transcripts are important for the interpretation of so much in biology. A key question is how we choose one single 'primary' transcript for each gene. These might be useful as default transcripts for displays, for variant effects, for comparative genomics etc. Choosing a 'primary' transcript for each gene could be done on the basis of coding sequence content, expression levels, clinical variant reporting, historical usage. Given the broad use of the transcripts, we would like your feedback for the impact on your work and to discover what different communities want in these transcript sets.

The two global sources of transcript annotation (RefSeq and Ensembl/GENCODE) will take your responses into account when formulating future strategies and resources.

This is a one page survey in four sections. It should take about 5-10 minutes to complete. The examples we use in the survey are all based on scenarios we frequently encounter during our curation.

Section 1 - Transcript choice (5 questions)
Section 2 - Variant interpretation and reporting (3 questions)
Section 3 - Reference sequence sources (2 questions)
Section 4 - About you

Next

Page 1 of 2

Never submit passwords through Google Forms.

This form was created inside EBI. Report Abuse

Section 1 - Transcript choice

Question 1)

Considering the transcripts of a gene, for your work how important is it to have:

- A minimal set of transcripts to cover ALL EXONS with evidence of CLINICAL SIGNIFICANCE A larger set of ALL known transcripts
- A minimal set of transcripts to cover
- ALL ABUNDANT PROTEIN-CODING EXONS
- Only ONE primary transcript
- A minimal set of transcripts to cover
- ALL ABUNDANT EXONS
- All of the above

Rate each as ‘critical’, ‘nice to have’ or ‘not needed’.

786 responses

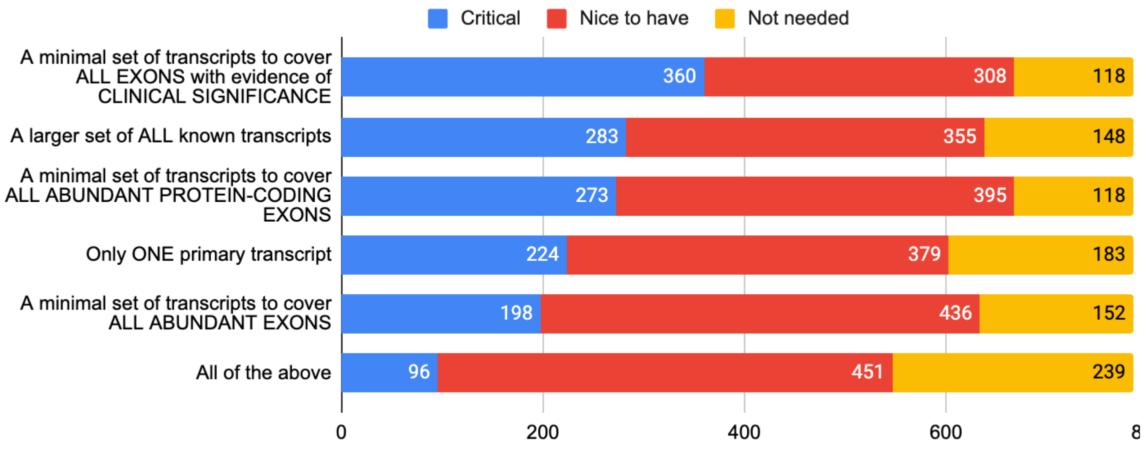

Other comments (N=106). Summary:

About a quarter of the comments expressed the importance of having the full set of transcripts per locus. A couple of respondents would prefer a theoretical transcript including all known exons, even if it wouldn't exist in nature. A similar number said it is useful to have a single most abundant transcript but then have any additional clinically important exons from other transcripts. Half the comments however, referred either to the fact that different transcript(s) are relevant to each situation (tissue type, tissue expressivity, cell-specificity, condition, environment, stage, abundance/quantity), or requested this type of information used as a rank or filter. More information was requested, for example on transcripts that were: computationally determined transcripts, predicted, fully-functional, validated, known to be functional, chosen by expert consensus as most clinically relevant, really rare. How non-protein-coding transcripts are determined or a primary transcript is determined.

Question 2a)
In the case of a gene WITHOUT any known clinically relevant variants, which transcript do you think should be the primary transcript (choose one)?

- The transcript that has the longest coding sequence (A)
- The transcript that is the most abundant (B)

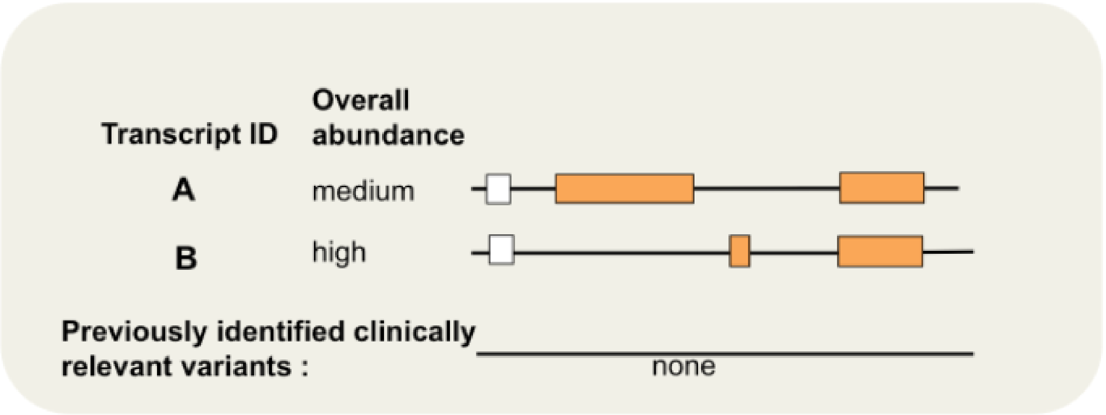

786 responses

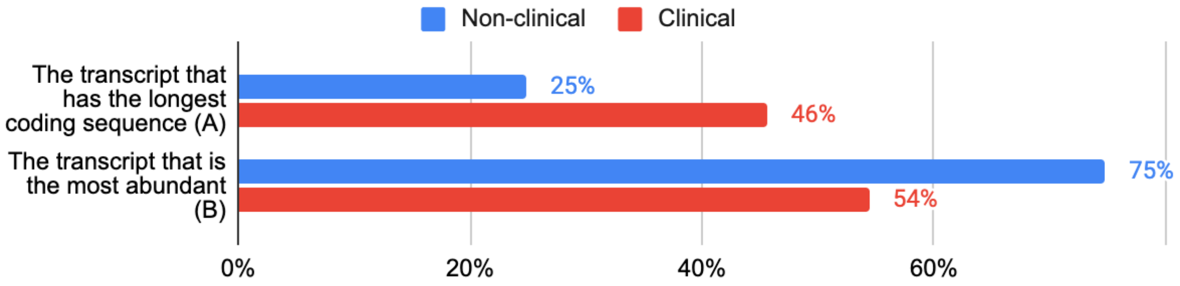

Additional comments (N=247). Summary:
There were a couple of comments expressing uncertainty on what to choose or that there should be no primary transcript defined if there are no clinically relevant variants. There were approximately 50 additional comments requesting that both transcripts were primary ones, or that abundance per tissue should be considered instead. Around 20 comments pointed out that abundance is hard to measure in a reliable and meaningful way, or that it can be altered in a disease state. 10% of comments said the primary transcript was a bad idea. Other comments suggested that the primary transcript should be a theoretical/virtual one to have all exons; be based on clinically relevant transcripts; be the longest; or be the most conserved.

Question 2b)
In the case of a gene WITHOUT any known clinically relevant variants, which transcript do you think should be the primary transcript (choose one)?

- The transcript that has the longest coding sequence (I)
- The transcript that is the most abundant (J)

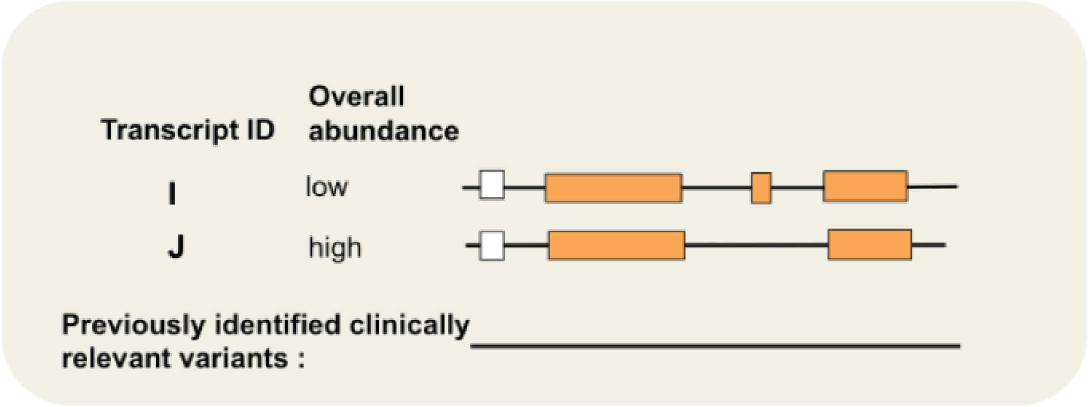

786 responses

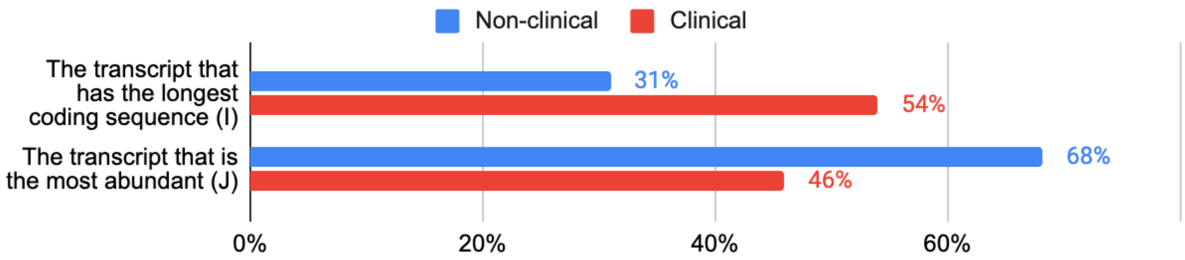

Additional comments (N=153). Summary:
Nearly all comments covered similar ground to question 2a. Some respondents reported current practice, without commenting on if this makes sense. Over 20 comments said both transcripts were required, and a similar number said that abundance is tissue-specific. A couple said that length was a better metric to use because abundance varies by tissue and this information is not always available / is open to interpretation, whereas length is defined. Others pointed out that the choice of transcript should be dependent on what is going on at the locus.

Question 3a)
In the case of a gene WITH clinically relevant variants, which transcript should be the single primary transcript (choose one)?

- Transcript that has the longest coding sequence (C)
- Transcript that covers the most clinically relevant variants (D)
- Transcript that is the most abundant (E)
- Transcript that has been most used historically

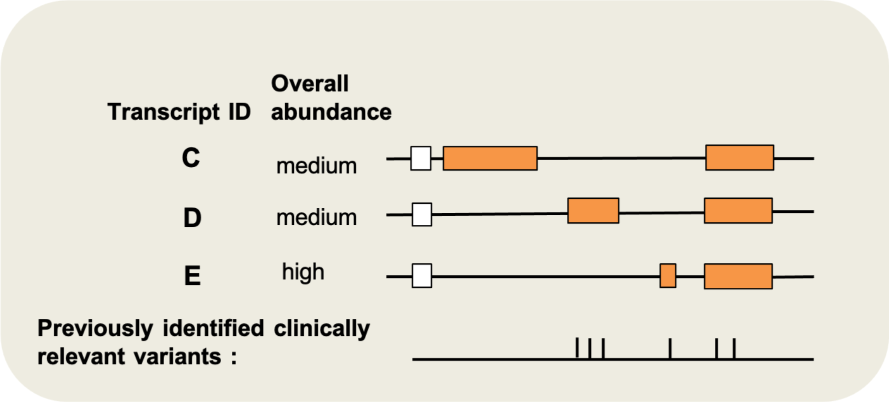

786 responses

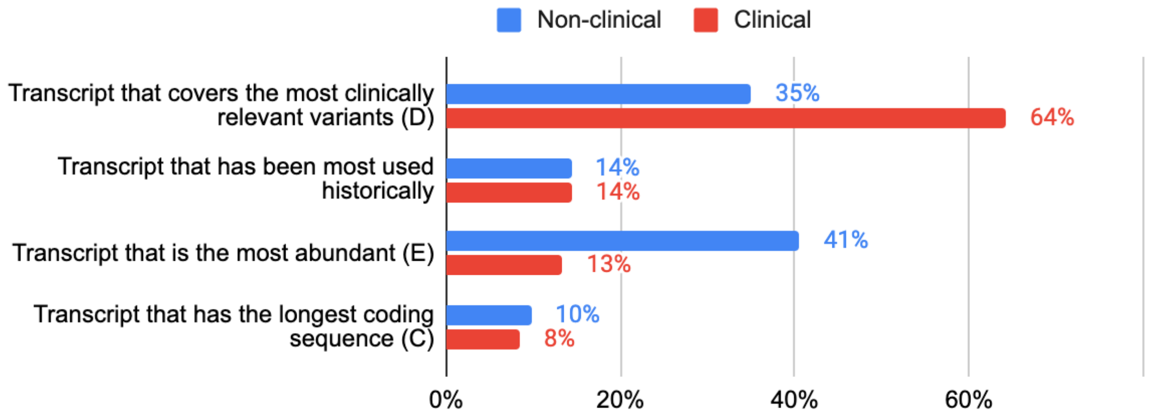

Additional comments (N=186). Summary:
Similar to in question 2, there were (about 10% each) comments that said use: all transcripts; two transcripts; no primary transcript; or a fake transcript created to contain all exons even if it has not been observed in nature. There were comments on the possible shortcomings of choosing one transcript or the difficulties of choosing one from this scenario. Some comments said that we should expect the primary transcript to change as new

clinically relevant variants are found or for other reasons; others question if the clinical variants can be trusted, or raised that they will change; others say it is important that there is no change as it is confusing, that there should be a consensus. There are comments that a ranking system should be available so everyone can prioritise to suit them, or that the oldest transcript should be used, or to choose the one that is most used in the literature; others say history needs to be abandoned because ‘so much literature lacks genomic coordinates’.

Question 3b)

In the case of a gene WITH clinically relevant variants, which transcript should be the single primary transcript (choose one)?:

- Transcript that is the most abundant overall (K)
- Transcript that is most abundant in the tissue of clinical relevance (L)
- Transcript that has been most used historically

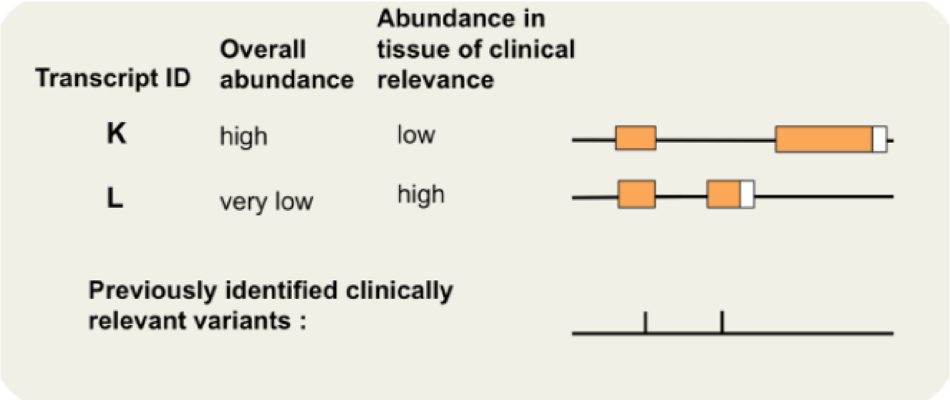

786 responses:

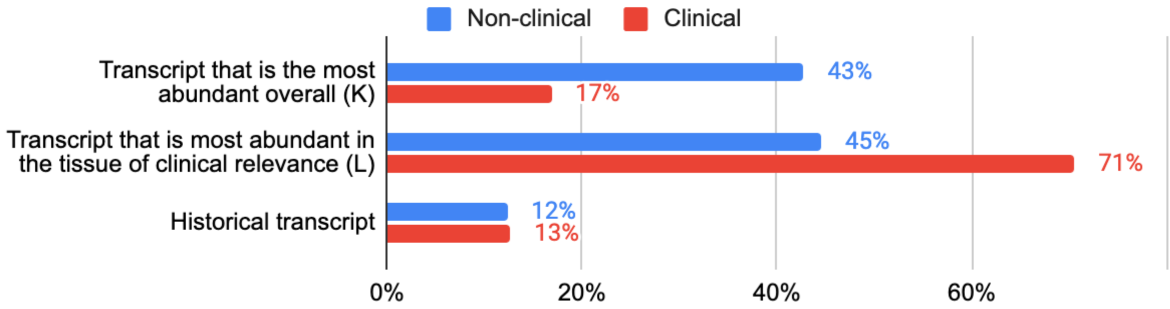

Additional comments (N=117). Summary

About a quarter of the comments either said to use both transcripts or that none should be chosen as primary. The others suggested to use the oldest, longest, a tissue-specific transcript or collapse all exons into a theoretical transcript. Several comments pointed out

that defining ‘clinically relevant’ is tricky as it will depend on the phenotype, developmental stage, cell, tissue, time, what you assay etc.

Question 4)

Considering the sequence of a transcript, which is the most important to you (choose one):

- That the sequence matches the reference assembly sequence (e.g. GRCh37/ hg19), even if it contains minor alleles
- That the sequence does not contain any pathogenic alleles
- That the sequence matches the global major allele
- That the sequence does not change
- It doesn’t matter to me

777 responses - only one answer allowed:

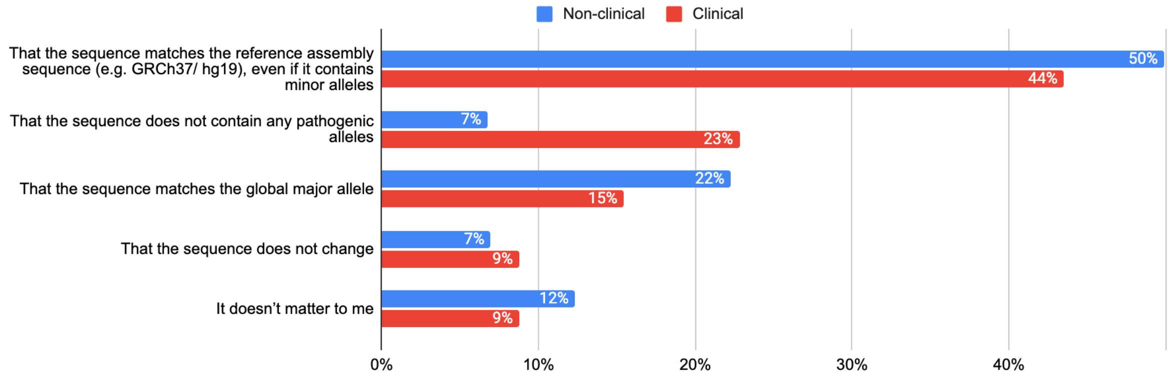

Question 5)

For your work, when is it appropriate to make an update to the primary transcript (select all that apply):

- A change in coding Sequence
- A change in UTR length
- A change of transcript splicing
- Never update

773 responses

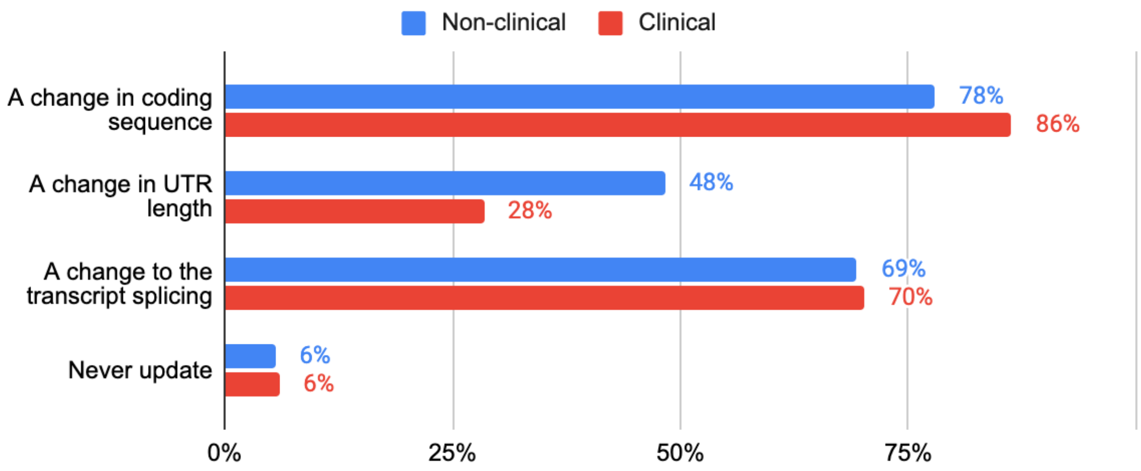

Optional comments (N=90). Summary:
Many comments referring to different interpretations of change. Not many were totally against any change and the vast majority accepted that change is required with several stressing importance of versioning. Mix of reasons for a change e.g. assembly change, function/abundance, clinical relevance. Overall impression given was a desire for being given the reason why annotation has changed e.g. new evidence, new biology etc.

Section 2 - Variant interpretation and reporting

Question 6)
If there is one primary transcript per locus, would you (check all that apply):

- Use it, and only it, for INTERPRETING the consequence of variants
- I wouldn't use just one transcript for INTERPRETATION unless it was the only one known
- Other

765 responses

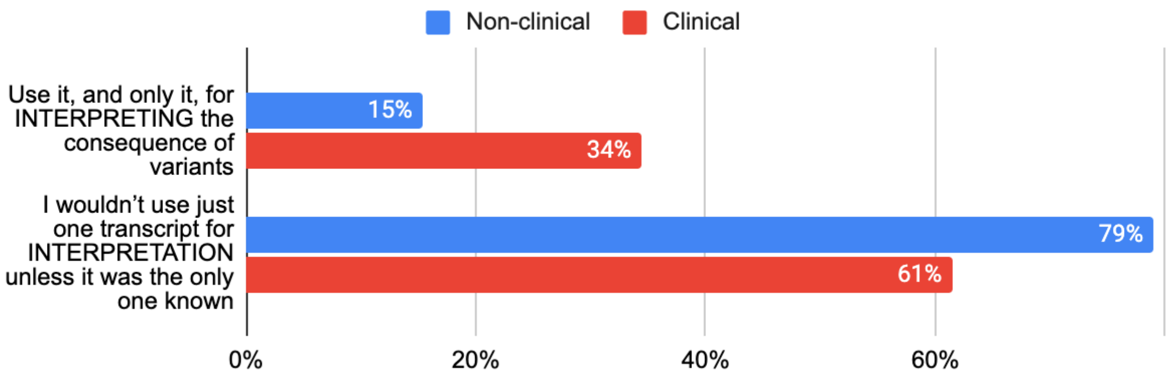

Other comments (N=74):
Comments for using: primary transcript if clinically relevant, all transcripts, a default but also looking at others as necessary, primary if tissue specific. Choices are dependent on the gene and if in a research or diagnostic setting.

Question 7)
If the most severe variant effect to be reported is not on the selected primary transcript (F), would you (choose one):

- Report the variant on the selected primary transcript (F) only
- Report the variant on the affected transcript (G) only
- Report the variant on both the selected primary transcript (F) and the affected transcript (G)
- Report the variant on all transcripts (F, G, H)
- I don't know
- Other

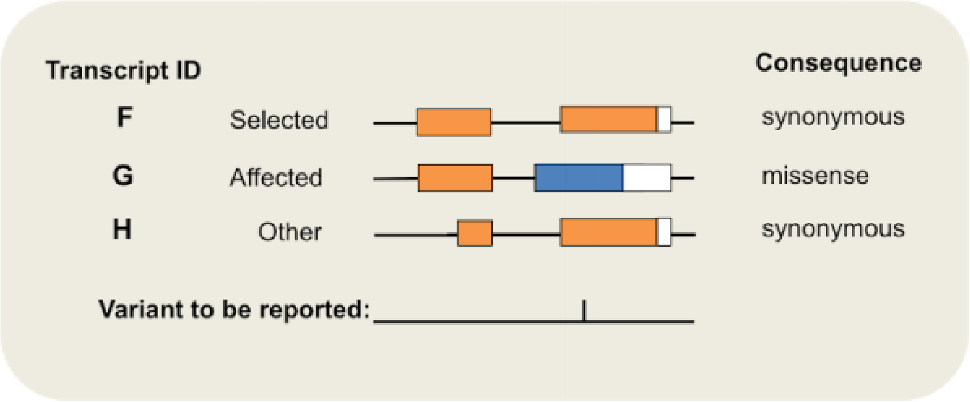

776 responses - only one response allowed

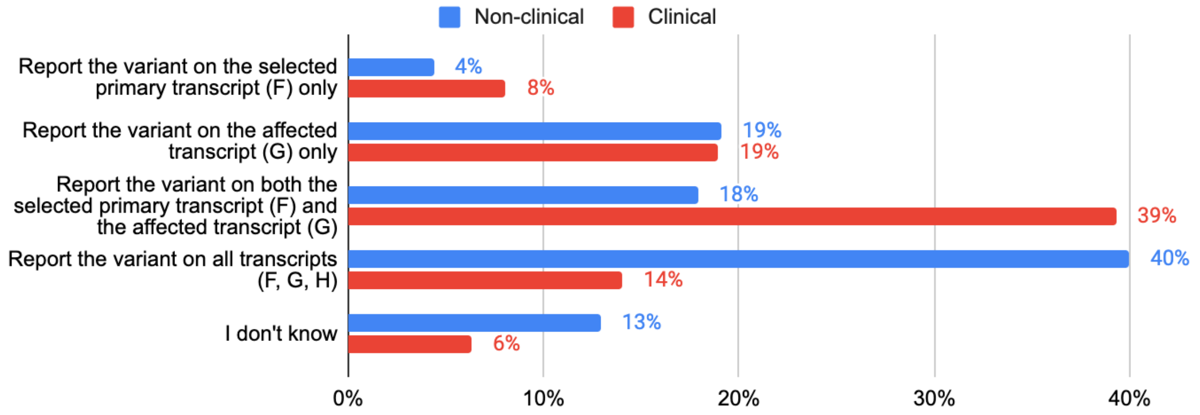

Question 8)

Which reference sequences do you use for reporting variants (select all that apply):

- RefSeq transcripts or proteins
- Ensembl/GENCODE transcripts or proteins
- GRCh37/hg19 genome
- GRCh38/hg38 genome
- LRG transcripts or LRG proteins

761 responses

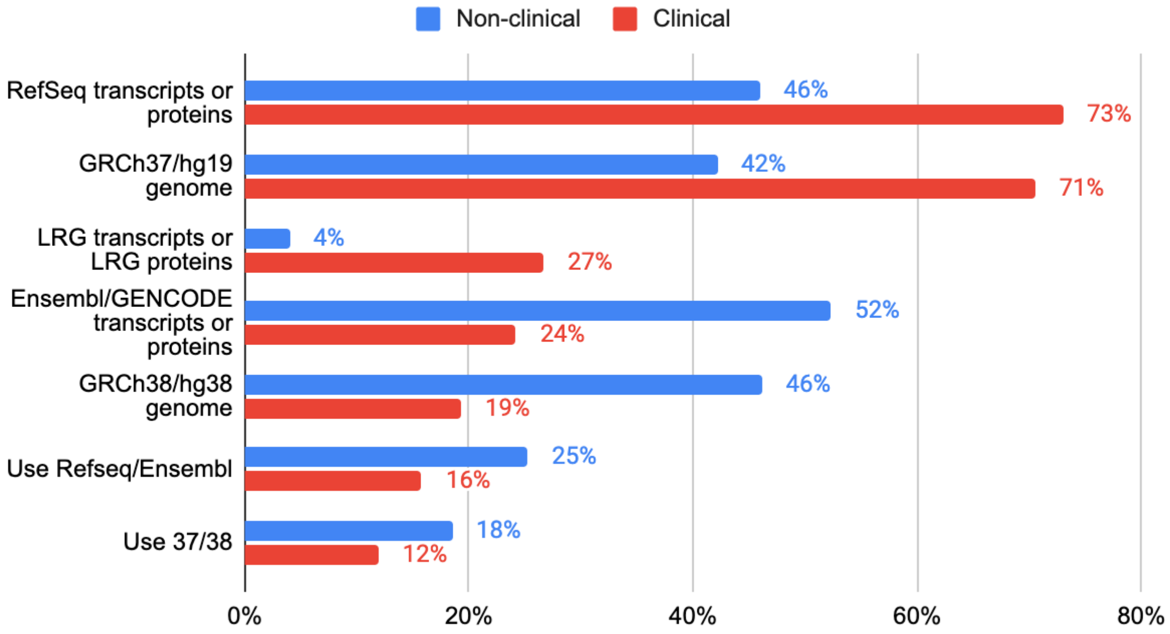

Section 3 - Reference sequence sources

Question 9)
Tick all that you believe are true:
- I use Ensembl/GENCODE transcripts for my work
- I use RefSeq transcripts for my work
- Both RefSeq and Ensembl/GENCODE transcripts are useful for my work
- I do not know whether RefSeq or Ensembl/GENCODE produce the best transcripts for my work
- I do not use RefSeq
- I do not use Ensembl/GENCODE

780 responses:

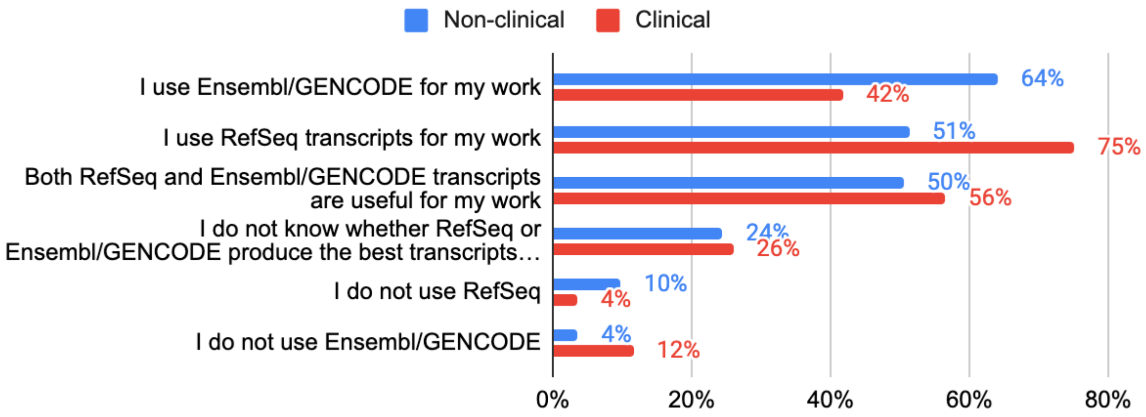

Question 10)
What is most important to you (select one):
- Having RefSeq and Ensembl/GENCODE agree on one primary transcript per gene
- Having different sets as they have different strengths
- I don't mind
- Other

775 Responses:

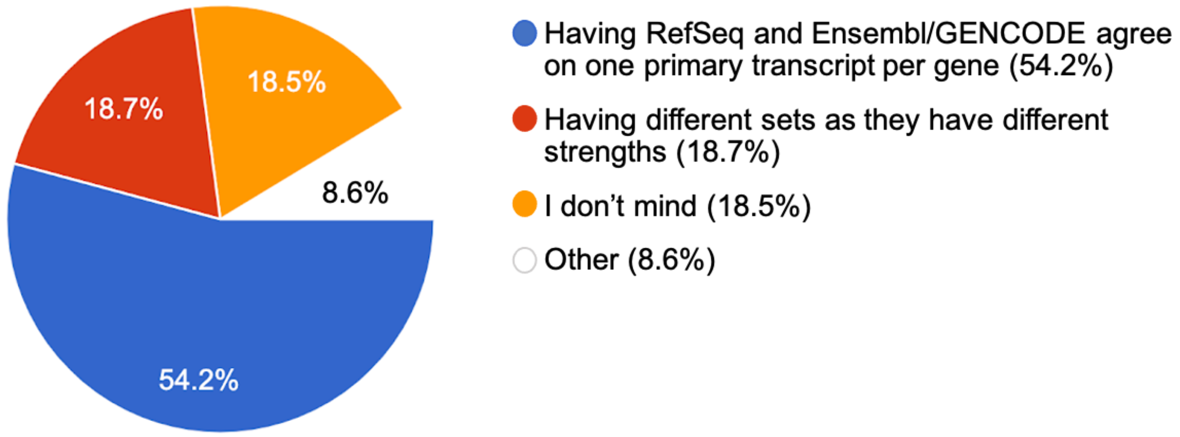

Pie chart for aggregated results above.

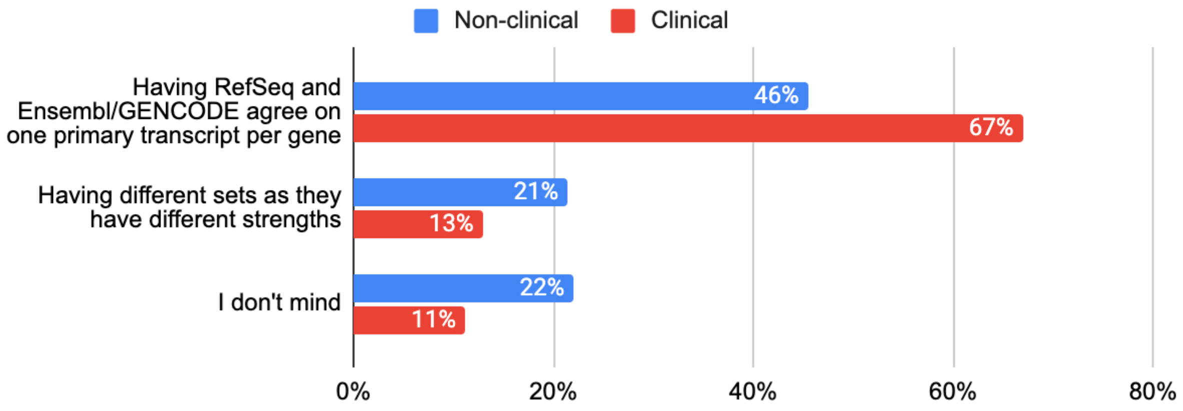

Bar chart for results split by clinical vs non-clinical respondent above.

Other comments (N=69). Summary:
Comments positive to having agreement, except for a few outliers who disagree with the whole idea of having a primary transcript. Very few use both sets currently. Perception that Ensembl has more transcripts than RefSeq, so RefSeq “simpler”.

Section 4 - About you

Question 11)
Which professional categories best describe you (select all that apply)?

782 responses.

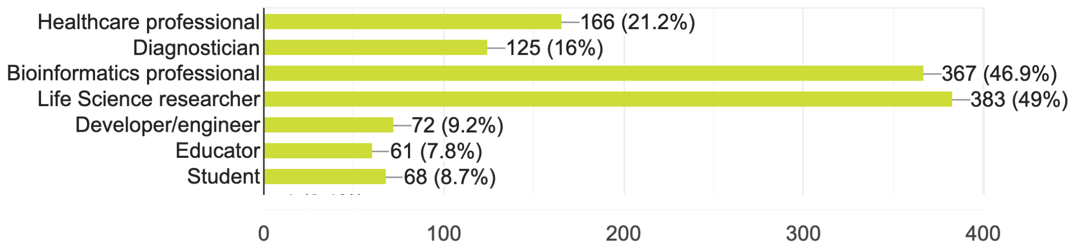

Question 12) Where do you work?

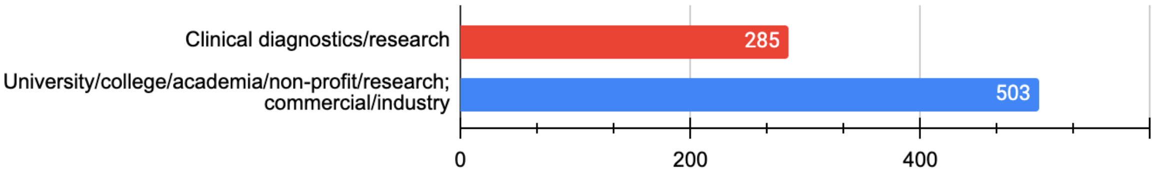

Question 13) In which country do you work?
767 responses

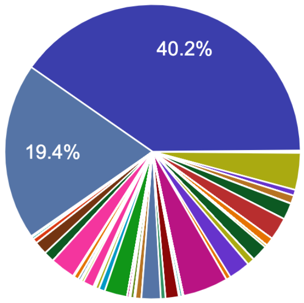

The survey generated 788 responses from 32 different countries: the top contributions were 40.2% from the US, 19.4% from the UK and 5% from Germany.

Question 14) Please briefly describe how you use transcripts
532 responses

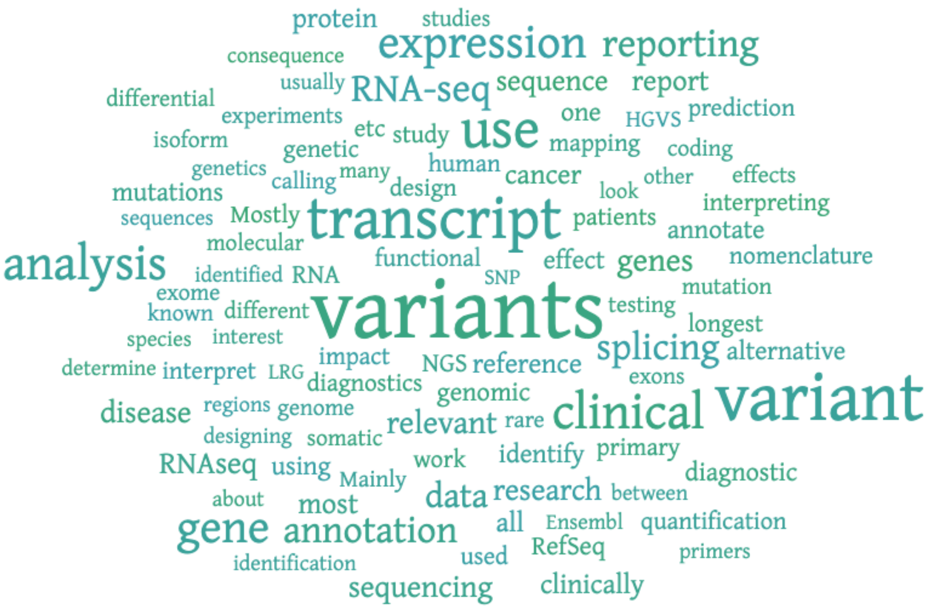

Word cloud above summarises the responses. The most frequently used words are larger.

Question 1 revisited)
Do you want us to provide one primary transcript?

776 responses

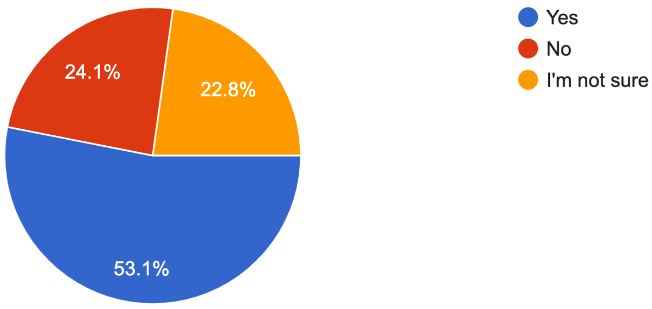

Pie chart for aggregated results

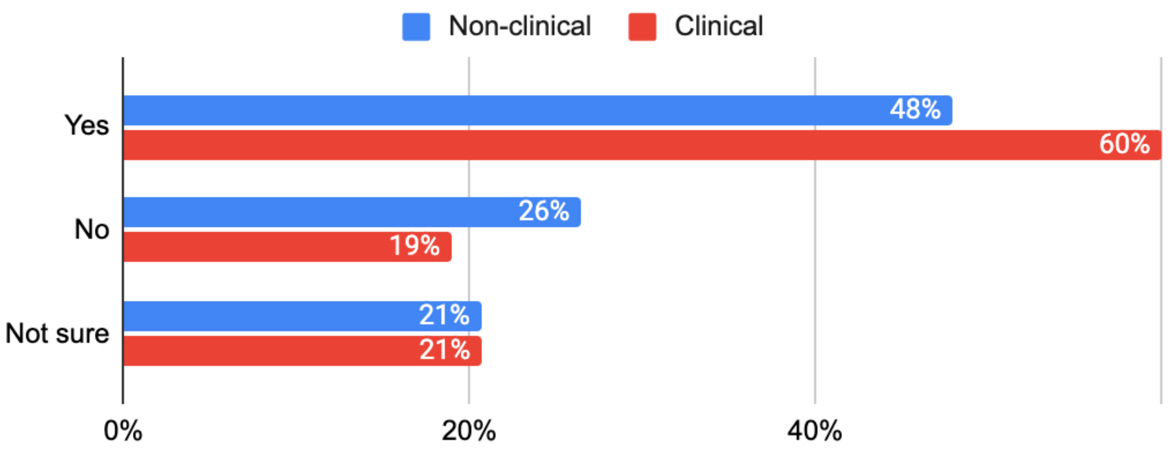

Bar chart for results split by clinical vs non-clinical respondent.

Additional information

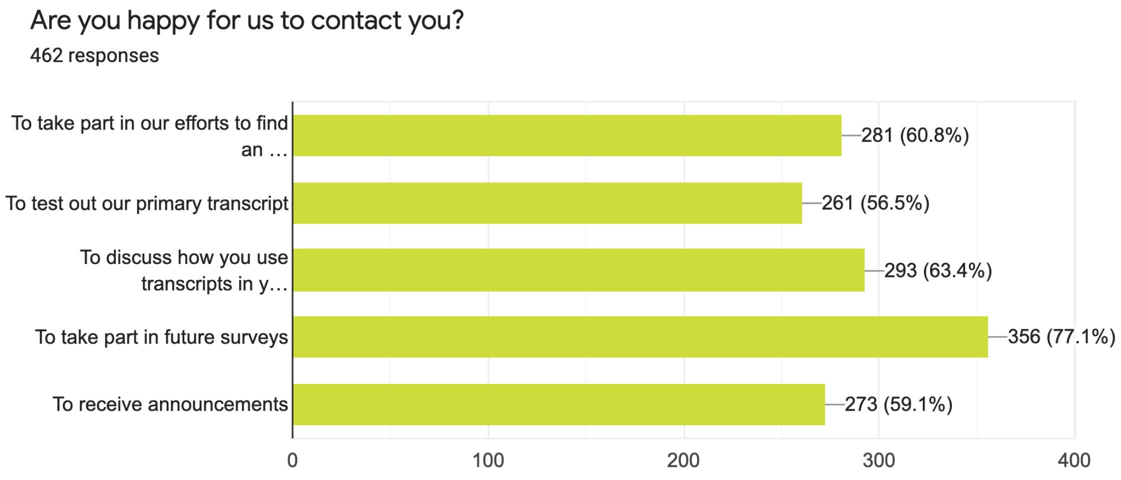

Multi-select answer options:

- To take part in our efforts to find an agreed, primary transcript;
- To test out our primary transcript;
- To discuss how you use transcripts in your work;
- To take part in future surveys;
- To receive announcements;
