# Supplementary material for: The value of primary transcripts to the clinical and non‐clinical genomics community: Survey results and roadmap for improvements
Source: Mol Genet Genomic Med. 2021 Aug 26;9(12):e1786. doi: 10.1002/mgg3.1786 (PMC8683622; doi:10.1002/mgg3.1786)
Supplement: Supplementary file 2 — Supplementary Material [file MGG3-9-e1786-s002.pdf]

Transcript Survey (one page)

Genes/transcripts are important for the interpretation of so much in biology. A key question is how we choose one single 'primary' transcript for each gene. These might be useful as default transcripts for displays, for variant effects, for comparative genomics etc. Choosing a 'primary' transcript for each gene could be done on the basis of coding sequence content, expression levels, clinical variant reporting, historical usage. Given the broad use of the transcripts, we would like your feedback for the impact on your work and to discover what different communities want in these transcript sets.

The two global sources of transcript annotation (RefSeq and Ensembl/GENCODE) will take your responses into account when formulating future strategies and resources.

This is a one page survey in four sections. It should take about 5-10 minutes to complete. The examples we use in the survey are all based on scenarios we frequently encounter during our curation.

Section 1 - Transcript choice (5 questions)
Section 2 - Variant interpretation and reporting (3 questions)
Section 3 - Reference sequence sources (2 questions)
Section 4 - About you

Next

Page 1 of 2

Never submit passwords through Google Forms.

This form was created inside EBI. Report Abuse

Transcript Survey (one page)

*Required

Untitled section

Section 1 - Transcript choice

1) Considering the transcripts of a gene, for your work how important is it to have: *

	Critical	Nice to have	Not needed
Only ONE primary transcript	○	○	○
A minimal set of transcripts to cover ALL EXONS with evidence of CLINICAL SIGNIFICANCE	○	○	○
A minimal set of transcripts to cover ALL ABUNDANT PROTEIN-CODING EXONS	○	○	○
A minimal set of transcripts to cover ALL ABUNDANT EXONS	○	○	○
A larger set of ALL known transcripts	○	○	○
All of the above	○	○	○

Additional comments (optional):

Your answer

2a) In the case of a gene WITHOUT any known clinically relevant variants, which transcript do you think should be the primary transcript? *

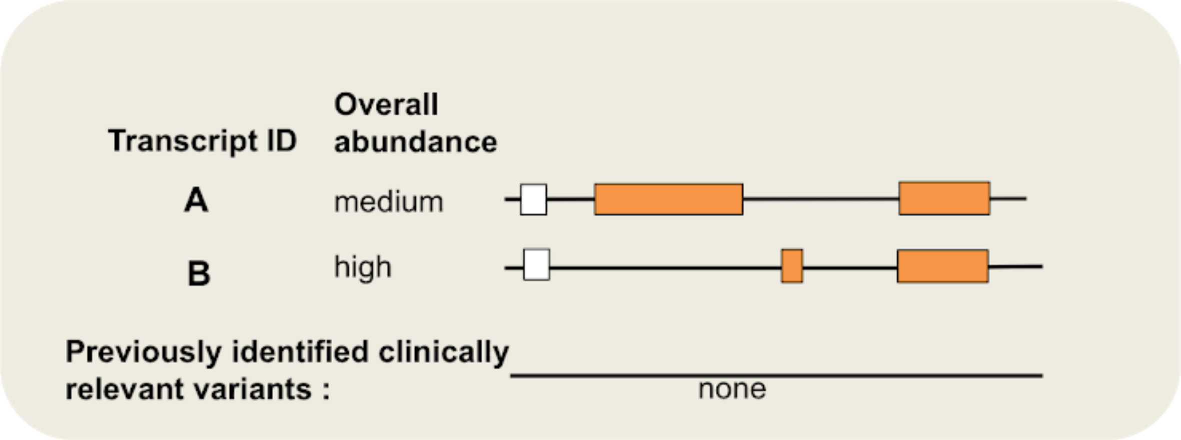

First example scenario

- The transcript that has the longest coding sequence (A)
- The transcript that is the most abundant (B)

Additional comments (optional)

Your answer

2b) In the case of a gene WITHOUT any known clinically relevant variants, which transcript do you think should be the primary transcript? *

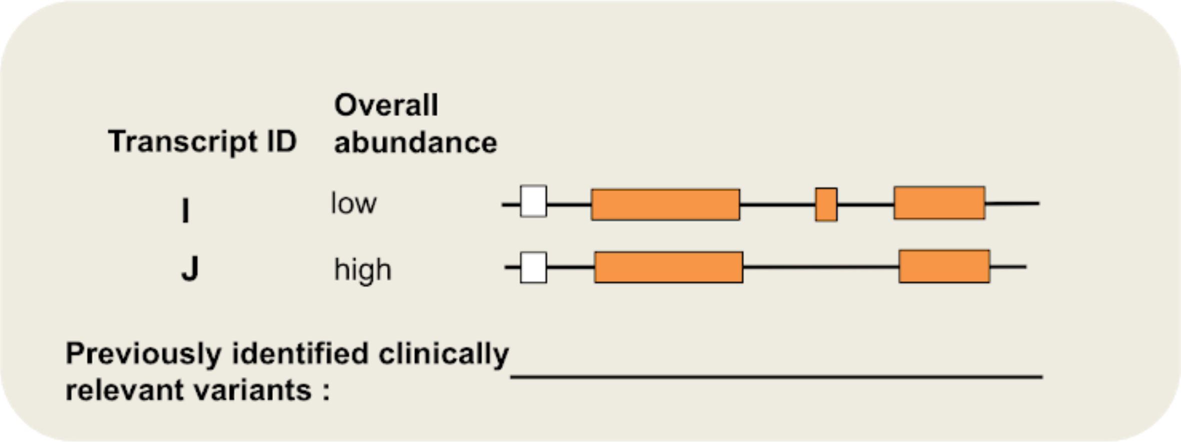

Second example scenario

- The transcript that has the longest coding sequence (I)
- The transcript that is the most abundant (J)

Additional comments (optional)

Your answer

3a) In the case of a gene WITH clinically relevant variants, which transcript should be the single primary transcript? *

Please answer based on which is best for your work.

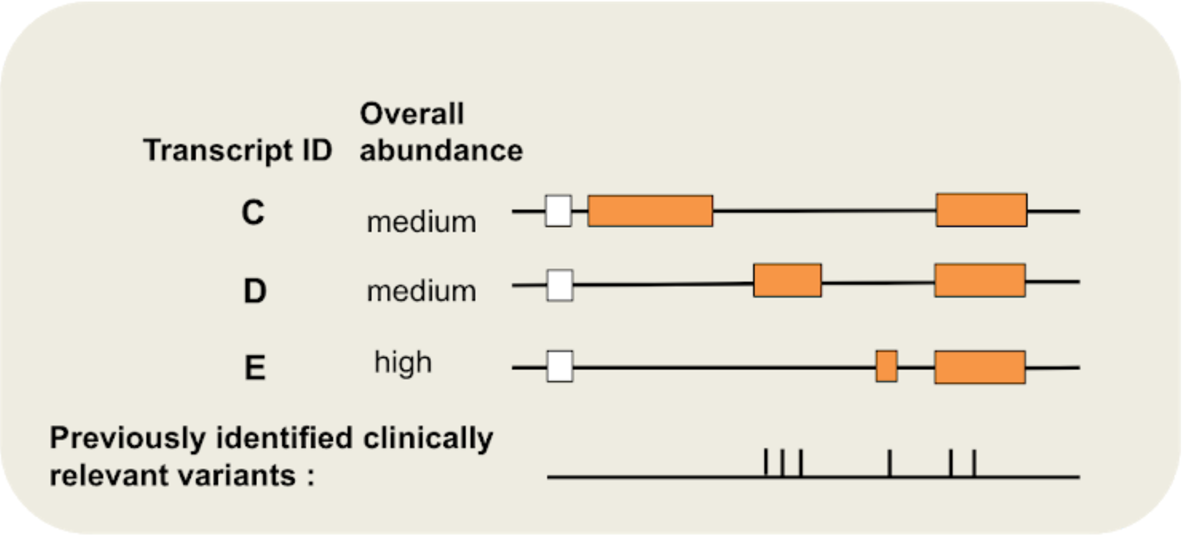

Third example scenario

- Transcript that has the longest coding sequence (C)
- Transcript that covers the most clinically relevant variants (D)
- Transcript that is the most abundant (E)
- Transcript that has been most used historically

Additional comments (optional)

Your answer

3b) In the case of a gene WITH clinically relevant variants, which transcript should be the single primary transcript? *

Please answer based on which is best for your work.

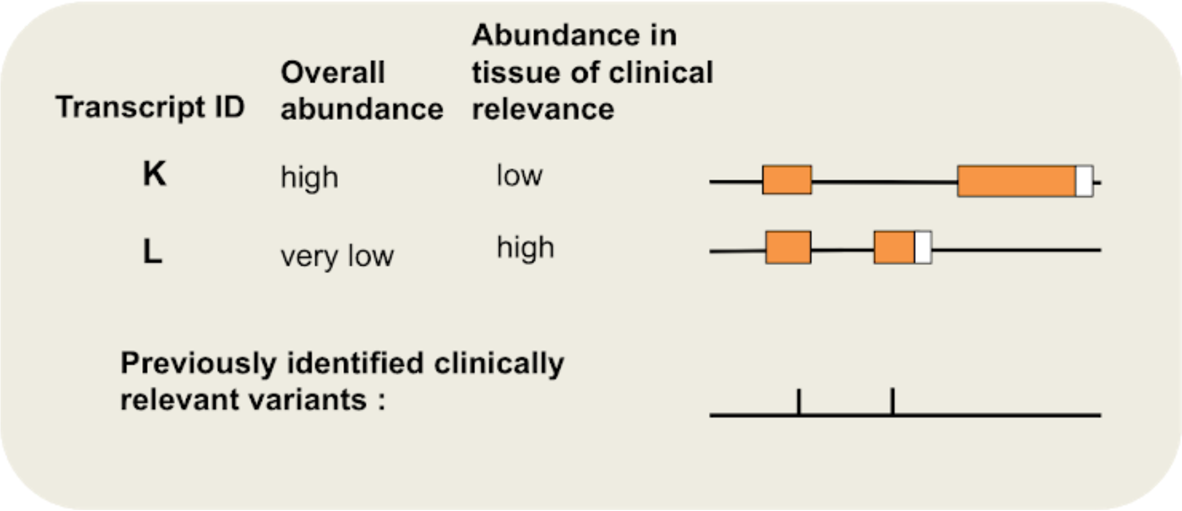

Fourth example scenario

- ○ Transcript that is the most abundant overall (K)
- ○ Transcript that is most abundant in the tissue of clinical relevance (L)
- ○ Transcript that has been most used historically

Additional comments (optional)

Your answer

4) Considering the sequence of a transcript, which is the most important to you:

- ○ That the sequence matches the reference assembly sequence (e.g. GRCh37/ hg19), even if it contains minor alleles
- ○ That the sequence does not contain any pathogenic alleles
- ○ That the sequence matches the global major allele
- ○ That the sequence does not change
- ○ It doesn't matter to me

5) For your work, when is it appropriate to make an update to the primary transcript:

Check all that apply

- [ ] A change in coding sequence
- [ ] A change in UTR length
- [ ] A change to transcript splicing
- [ ] Never update
- [ ] Other: ____________________

Section 2 - Variant interpretation and reporting

6) If there is one primary transcript per locus, would you:

Check all that apply

- [ ] Use it, and only it, for INTERPRETING the consequence of variants
- [ ] I wouldn't use just one transcript for INTERPRETATION unless it was the only one known
- [ ] Other: ____________________

7) If the most severe variant effect to be reported is not on the selected primary transcript (F), would you:

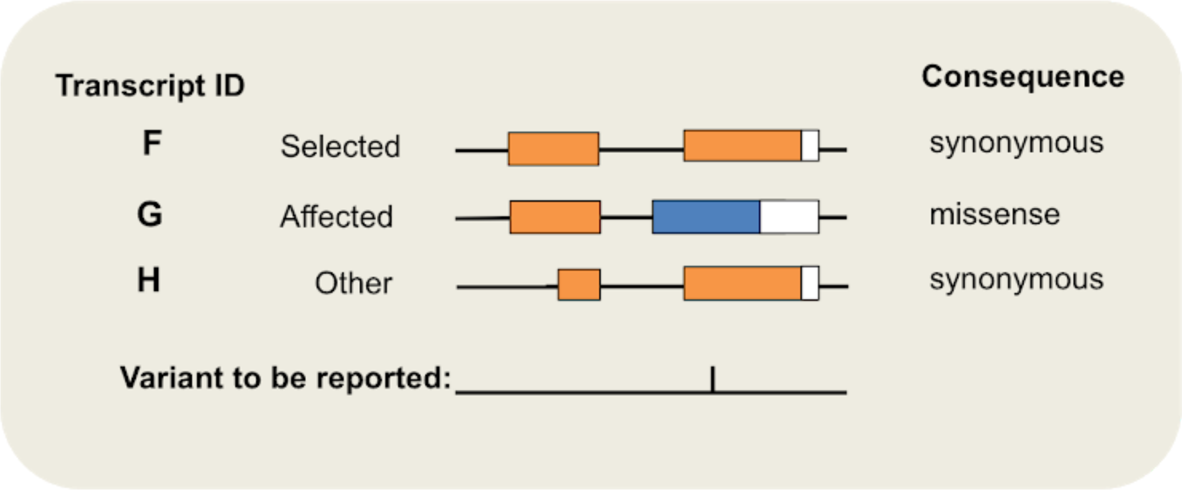

Fourth example scenario

Blue indicates that the second exon of transcript G is in a different frame than F and H, and codes for a unique protein (different C-terminal peptide tail).

- ◯ Report the variant on the selected primary transcript (F) only
- ◯ Report the variant on the affected transcript (G) only
- ◯ Report the variant on both the selected primary transcript (F) and the affected transcript (G)
- ◯ Report the variant on all transcripts (F, G, H)
- ◯ I don't know
- ◯ Other: ____________

8) Which reference sequences do you use for reporting variants:

Check all that apply

- ☐ RefSeq transcripts or proteins
- ☐ Ensembl/GENCODE transcripts or proteins
- ☐ GRCh37/hg19 genome
- ☐ GRCh38/hg38 genome
- ☐ LRG transcripts or LRG proteins

Section 3 - Reference sequence sources

9) Tick all that you believe are true:

- [ ] I use RefSeq transcripts for my work
- [ ] I do not use RefSeq
- [ ] I use Ensembl/GENCODE transcripts for my work
- [ ] I do not use Ensembl/GENCODE
- [ ] Both RefSeq and Ensembl/GENCODE transcripts are useful for my work
- [ ] I do not know whether RefSeq or Ensembl/GENCODE produce the best transcripts for my work

10) What is most important to you:

- ○ Having RefSeq and Ensembl/GENCODE agree on one primary transcript per gene
- ○ Having different sets as they have different strengths
- ○ I don’t mind
- ○ Other: ______

Additional comments (optional):

Your answer ______

Section 4 - About you

11) Which professional categories best describe you?

Check all that apply

- [ ] Healthcare professional
- [ ] Diagnostician
- [ ] Bioinformatics professional
- [ ] Life Science researcher
- [ ] Developer/engineer
- [ ] Educator
- [ ] Student
- [ ] Other: ____________________

12) Where do you work?

Check all that apply

- [ ] University/College/Academia/Non-profit/Research
- [ ] Clinical diagnostics
- [ ] Clinical research
- [ ] Commercial/Industry
- [ ] Government
- [ ] Other: ____________________

13) In which country do you work?

Choose ▾

14) Please briefly describe how you use transcripts

Your answer

Question 1 revisited - a primary transcript

Do you want us to provide one primary transcript?

- ◯ Yes
- ◯ No
- ◯ I'm not sure

Additional comments (optional)

Your answer

Contact

If you are willing to be contacted, please leave your details. We will add you to a mailing list; you can unsubscribe at any time.

Are you happy for us to contact you?

Check all that apply

- ☐ To take part in our efforts to find an agreed, primary transcript
- ☐ To test out our primary transcript
- ☐ To discuss how you use transcripts in your work
- ☐ To take part in future surveys
- ☐ To receive announcements

Name

Your answer

Email Address

Your answer

Affiliation

Your answer

More information
http://www.lrg-sequence.org/sites/lrg-sequence.org/files/documents/lrg_ref_seq_source.pdf

Page 2 of 2

Back

Submit

Never submit passwords through Google Forms.

This form was created inside EBI. Report Abuse

Google Forms
